# Single Administration of p*2TA* (AB103), a CD28 Antagonist Peptide, Prevents Inflammatory and Thrombotic Reactions and Protects against Gastrointestinal Injury in Total-Body Irradiated Mice

**DOI:** 10.1371/journal.pone.0101161

**Published:** 2014-07-23

**Authors:** Salida Mirzoeva, Tatjana Paunesku, M. Beau Wanzer, Anat Shirvan, Raymond Kaempfer, Gayle E. Woloschak, William Small

**Affiliations:** 1 Department of Radiation Oncology, Northwestern University Feinberg School of Medicine, Chicago, Illinois, United States of America; 2 Atox Bio Ltd, Ness Ziona, Israel; 3 Department of Biochemistry and Molecular Biology, Institute of Medical Research Israel-Canada, The Hebrew University-Hadassah Medical School, Jerusalem, Israel; 4 Department of Radiation Oncology, Loyola University Stritch School of Medicine, Chicago, Illinois, United States of America; National Cancer Institute, United States of America

## Abstract

The goal of this study was to elucidate the action of the CD28 mimetic peptide p*2TA* (AB103) that attenuates an excessive inflammatory response in mitigating radiation-induced inflammatory injuries. BALB/c and A/J mice were divided into four groups: Control (C), Peptide (P; 5 mg/kg of p*2TA* peptide), Radiation (R; total body irradiation with 8 Gy γ-rays), and Radiation + Peptide (RP; irradiation followed by p*2TA* peptide 24 h later). Gastrointestinal tissue damage was evaluated by analysis of jejunum histopathology and immunohistochemistry for cell proliferation (Cyclin D1) and inflammation (COX-2) markers, as well as the presence of macrophages (F4/80). Pro-inflammatory cytokines IL-6 and KC as well as fibrinogen were quantified in plasma samples obtained from the same mice. Our results demonstrated that administration of p*2TA* peptide significantly reduced the irradiation-induced increase of IL-6 and fibrinogen in plasma 7 days after exposure. Seven days after total body irradiation with 8 Gy of gamma rays numbers of intestinal crypt cells were reduced and villi were shorter in irradiated animals compared to the controls. The p*2TA* peptide delivery 24 h after irradiation led to improved morphology of villi and crypts, increased Cyclin D1 expression, decreased COX-2 staining and decreased numbers of macrophages in small intestine of irradiated mice. Our study suggests that attenuation of CD28 signaling is a promising therapeutic approach for mitigation of radiation-induced tissue injury.

## Introduction

Exposure to ionizing radiation (IR) promotes both inflammatory reactions and immune system dysbalance. Radiation-induced acute inflammatory responses have been shown to activate multiple pro-inflammatory cytokines and inhibit anti-inflammatory cytokines; thus, cytokines are often used to modulate the effects of IR [1]. The excessive gastrointestinal (GI) inflammatory response that occurs following radiation is considered one of the drivers of multiple organ failure induced by IR [2], [3]. For example, pulmonary injury may be an abscopal effect of GI irradiation injury [4]. Therefore, modulating radiation induced inflammatory reactions, especially in the GI tract can have significant effects on the rest of the organism.

Cluster of differentiation 28 (CD28) antigen is expressed on T cells and is required for their activation as well as the survival and expansion of the peripheral blood T cells. Stimulation through CD28 can provide a potent co-stimulatory signal to T cells for the production of multitude of pro-inflammatory mediators, including IL-6 and fibrinogen, both involved in the progression of tissue injury. Moreover, T cells recruit peripheral macrophages to irradiated tissues [5], [6]. Short peptides can prevent CD28 signaling induced by superantigen toxins [7], [8] or streptococcal infection [9]. p*2TA* (also designated AB103) is an octapeptide mimetic of the CD28 homodimer interface that prevents the engagement of CD28 by superantigens *in* *vivo*, thus averting T cell activation leading to protection from lethal toxic shock caused by an excessive inflammatory response [8], [10]. At the same time, p*2TA* leaves the Th2-cytokine based humoral immune response intact [8], [9]. Because p*2TA* attenuates the CD28 cascade and inflammatory cytokine response, we hypothesized that it may be useful as a mitigator of radiation effects associated with inflammation.

Our objective in these studies was to evaluate the effect of the peptide p*2TA* in mice when administered 24 h after a total-body 8 Gy γ-ray dose. We evaluated systemic and tissue inflammatory responses in plasma, small intestine, lung, heart and spleen. The use of the peptide p*2TA* significantly decreased inflammatory responses and tissue injury 7 days after irradiation. This study suggests that the development of CD28-oriented therapeutic approaches for the treatment or prevention of radiation-induced inflammation could lead to important radioprotective and clinical benefits.

## Materials and Methods

### Ethics statement

All animal studies, housing and experiment were carried out with the Northwestern University Animal Care and Use Committee (IACUC) approval, permit number 2010-2178. Northwestern University has an Animal Welfare Assurance on file with the Office of Laboratory Animal Welfare (A3283-01) and conducts its reviews in accordance with United States Public Health Service (USPHS) regulations and applicable federal and local laws.

### Mice

Two inbred strains of mice, BALB/c and A/J, were selected for this study because of their differences in radiosensitivity [11]. While the LD50/30 in these two strains differs, an LD50/30 of 5.9 Gy for A/J and 5.7 Gy for BALBc mice according to [12], this difference was not likely to be translated into overwhelming cellular radiation response differences at day 7 after an exposure to a dose of 8 Gy. Eight week old BALB/c and A/J male mice were obtained from Jackson Laboratories (Bar Harbor, ME). Mice received rodent chow (Harlan Teklad, WI) and water *ad libitum*.

### Peptide

Peptide p*2TA* (amino acid sequence SPMLVAYD) covering residues 8–15 of the extracellular domain of CD28, bounded with D-Ala at both termini for greater protease resistance [7], [8], was synthesized using fluoronyl-methoxycarbonyl chemistry, cleaved and the side chain deprotected with trifluoroacetic acid. p*2TA* was >95% pure by high-pressure liquid chromatography; its molecular weight was verified by MALDI-TOF mass spectrometry.

### Irradiation

Eight mice of each strain were given a single total-body radiation dose of 8 Gy cesium ^137^Cs gamma rays, dose rate 95.7 cGy/min (Best Theratronics, Ottawa, Canada). Sham-irradiated animals were treated in the same manner but were not exposed to the source. Twenty-four hours later, half of the sham- and 8 Gy-irradiated mice were injected with p*2TA* (5 mg/kg) via tail vein injection. Irradiations and peptide treatments were done at the similar time of day, between 11 am and 2 pm. Animals were sacrificed by CO_2_ asphyxiation followed by cervical dislocation 7 days after irradiation (6 days after peptide injection).

### Measurement of pro-inflammatory mediators

Blood samples were collected immediately after sacrifice by intracardiac puncture. Concentration of IL-6 and KC in diluted plasma (1∶3) was determined using a specific ELISA kit (IL-6, eBioscience, San Diego, CA; KC, R&D Systems, Minneapolis, MN). Fibrinogen concentration determination in plasma samples diluted 1∶20,000 was done using a specific ELISA kit (Immunology Consultants Laboratory, Portland, OR). Recombinant mouse IL-6, KC and fibrinogen were used as standards.

### Immunohistochemistry

The mid-jejunum of small intestine, lung and spleen tissues were dissected, fixed in 10% buffered formalin, embedded in paraffin, and sectioned into 4 µm thin sections. Slides were stained by hematoxylin and eosin (H&E); immunohistochemistry was performed at the Northwestern University Mouse Histopathology Core Facility with in-house developed antibodies for with COX-2, Cyclin D1 and macrophage marker F4/80. Slides were imaged using a TissueGnostics microscope (Zeiss, Oberkochen, Germany). Quantification of the immunohistochemistry results was done with HistoQuest software.

### Histopathological analysis of small intestine

Intestinal damage was assessed by measurements of villus height and the numbers of surviving crypts in each circumference using H&E-stained jejunal slides. Villus height was measured from the bottom of the crypt to the crypt-villus junction. The number of surviving crypts was determined using the criteria that a surviving crypt must contain at least 10 epithelial cells, at least one Paneth cell and a lumen [13]. Four circumferences were scored per mouse in each group. The viability of surviving crypts was confirmed by immunohistochemical detection of Cyclin D1.

### Data analysis

Data from all animals in the group were pooled and presented as means +/− SEM. Statistical analyses were performed by analysis of variance and the t-test. A P value of less than 0.05 indicated statistical significance.

## Results

### Animals

Eight animals each were exposed to 8 Gy of total-body irradiation; four of them were injected with p*2TA* peptide 24 h later in order to evaluate whether p*2TA* can mitigate the effects of radiation. The LD50/30 of A/J and BALB/c mice are 5.9 Gy and 5.7 Gy, respectively [12]. It could be expected that all mice exposed to 8 Gy should still be alive at 7 days post-radiation (6 days after peptide delivery). By choosing these timepoints we desired to allow the benefits of p*2TA* peptide to take effect and to be able to observe its effects on plasma markers (e.g. elevated fibrinogen levels are expected in the plasma at day 7 [14]. If these mice were more radioresistent, injury to jejunal mucosa would have been maximal at day 3.5 followed by recovery until day 21 after irradiation [15]; however, considering that LD50/30 of these mouse strains was lower than the dose delivered, it is likely that high percentage of animals would die before 21 days of age.

### Effect of p*2TA* peptide on plasma proteins following whole body gamma irradiation

**Figure 1:**
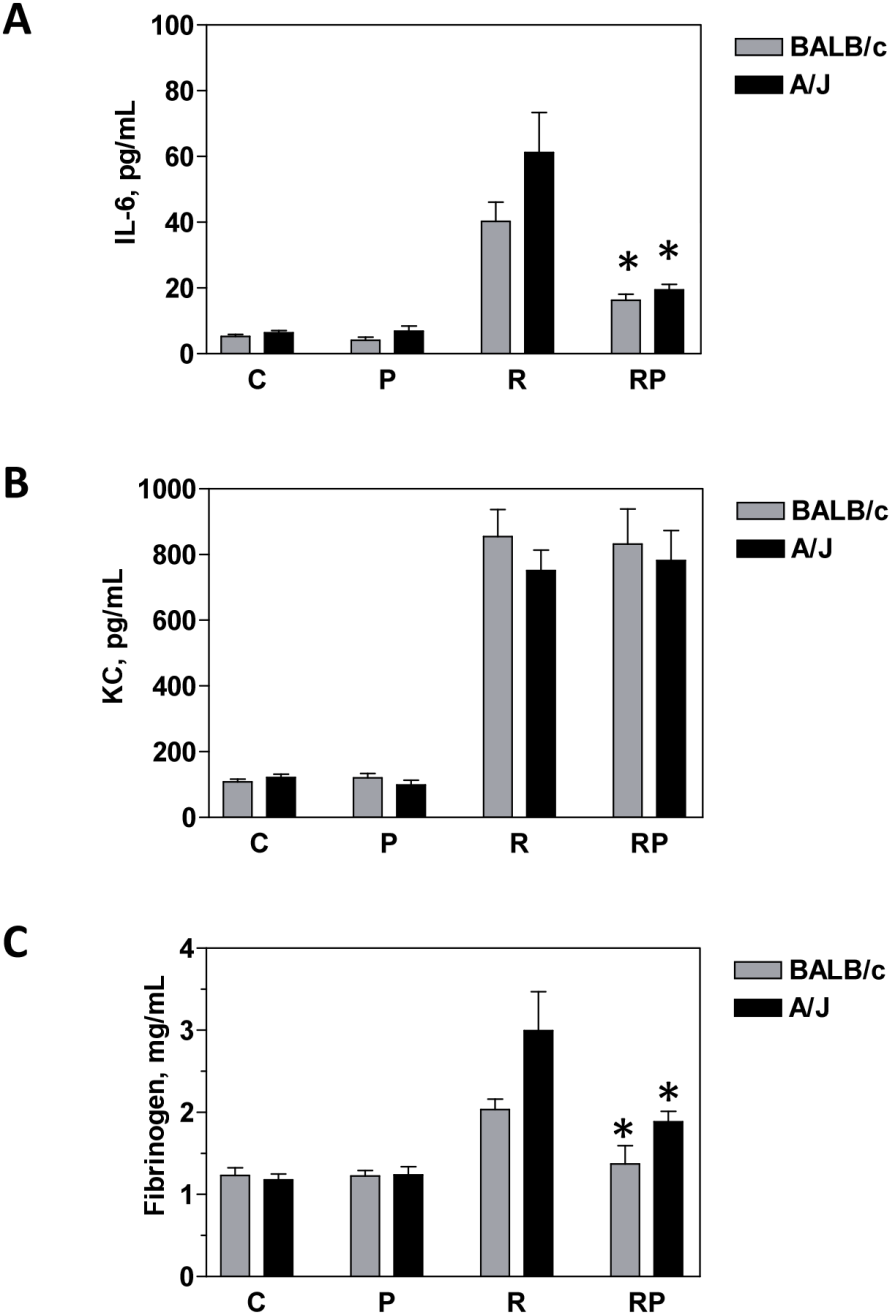
The effect of p*2TA* peptide on systemic inflammatory mediators. Plasma levels of IL-6 (panel **A**), KC (panel **B**) or fibrinogen (panel **C**) were measured on day 7 after irradiation. C: sham-irradiated mice, P: sham-irradiated mice that received 5 mg/kg of the p*2TA* peptide, R: 8 Gy-irradiated mice, RP: 8 Gy-irradiated mice that received 5 mg/kg of the p*2TA* peptide 24 h after irradiation. Bars represent the means ± SEM of 4–5 mice. *Statistically significant difference between R and RP groups (P<0.05).

**Cytokine interleukin 6 (IL-6)** is an important mediator of the acute phase of protein expression and hematopoietic cell production [16]. Seven days after radiation exposure, IL-6 had increased significantly in the plasma of irradiated mice (Figure 1A), as expected based on previous reports [14]. IL-6 levels were enhanced 6.9- and 8.9-fold, respectively, in BALB/c and A/J mice (P<0.05, R vs. C group). Injection of sham-irradiated mice with p*2TA* peptide did not affect IL-6 levels (Figure 1A). In contrast, treatment of irradiated mice with p*2TA* resulted in a significant reduction of IL-6, 40.4% in BALB/C and 31.8% in A/J mice compared to irradiated mice (P<0.05; PR vs. R group) (Figure 1A).

**Keratinocyte-derived chemokine (KC)** is a neutrophil and monocyte chemo-attractant [17]. As expected from previous reports [14], whole-body irradiation significantly increased KC plasma levels (Figure 1B). The KC level increased 4.0- and 3.4-fold in BALB/c and A/J mice, respectively (P<0.05, R group versus C group). Injection of sham-irradiated mice with p*2TA* slightly decreased KC plasma level in BALB/c and did not affect KC level in A/J mice (Figure 1B). In irradiated animals treated with p*2TA* peptide no change in BALB/c and some increase in A/J mice plasma was detected (Figure 1B). None of these changes were statistically significant.

**Fibrinogen** levels in plasma were elevated after radiation exposure, although that increase has been reported to be associated with bacterial infection [14], [18]. The fibrinogen levels (Figure 1C) in irradiated BALB/c and A/J mice were increased 1.6- and 2.5-fold, respectively, compared to sham-irradiated animals. The injection of sham-irradiated mice with p*2TA* peptide did not affect fibrinogen levels significantly. In irradiated mice a significant decrease in fibrinogen plasma levels was observed in mice treated with p*2TA*. Fibrinogen levels in irradiated and p*2TA*-treated mice were 67.5% (BALB/c) and 62.9% (A/J) of fibrinogen levels in irradiated counterparts (P<0.05; PR vs. R group) (Figure 1C).

### p*2TA* peptide protects small intestine from radiation-induced damage

**Figure 2:**
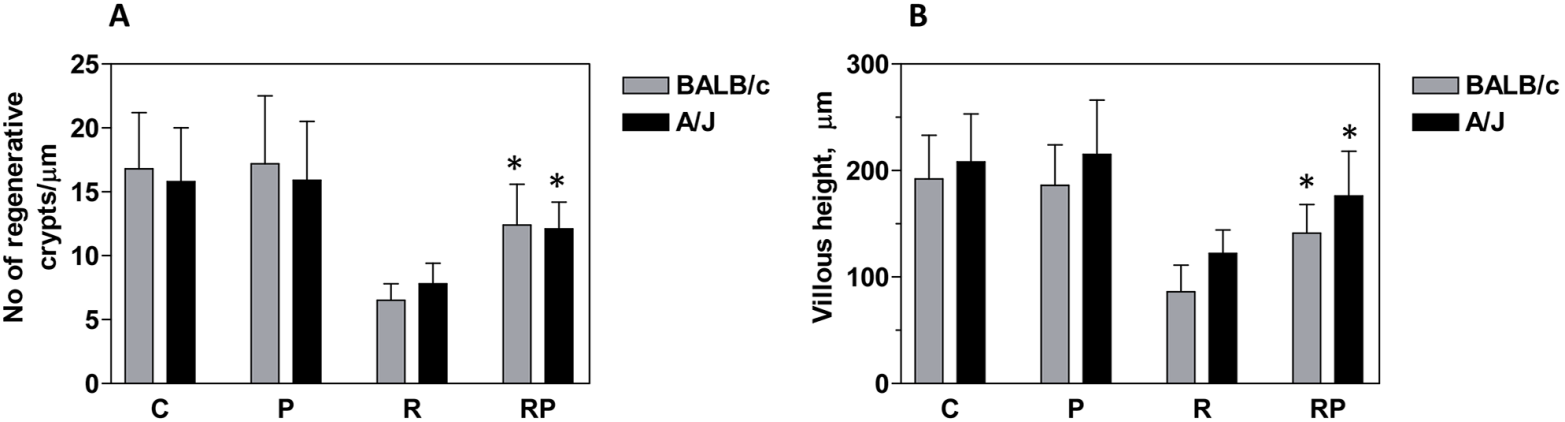
The effect of p*2TA* peptide on jejunal crypts. Jejunal tissues were collected 7 days after irradiation and embedded in paraffin. After fixation and processing, cross sections of jejunum were stained with hematoxylin and eosin and analyzed for the number of surviving crypts (panel **A**) and villus height (panel **B**). Bars represent the means ± SEM of 4 cross-sections for each mouse, and 4–5 mice in each group. *Statistically significant difference between R and RP groups (P<0.05).

**The number of jejunal crypts** following whole-body irradiation correlates with animal survival; we counted them on H&E stained slides following a defined protocol of Withers and Elkind [13] (Figure 2A). The number of crypt microcolonies was significantly higher in the intestines of radiation and p*2TA* peptide-treated (RP) mice compared with intestines from irradiated (R) mice. In BALB/c mice, the number of microcolonies increased from 6.5±1.3/µm in R group to 12.4±3.2/µm in RP group (P<0.05). In A/J mice, the number of microcolonies increased from 7.8±1.6/µm in R group to 12.1±2.1/µm in RP group (P<0.05).

**Villus height in jejunal cross sections** is another hallmark of GI injury (Figure 2B). Mean villus height was 192±41 µm for control BALB/c and 208±45 µm for control A/J mice. In irradiated mice, mean villus height decreased to 86±25 µm for BALB/c and 122±22 µm for A/J mice. Administration of p*2TA* peptide reduced some of this radiation-induced effect. The mean villus height in irradiated BALB/c and A/J mice treated with p*2TA* increased to 141±27 µm and 176±42 µm, respectively. p*2TA* had little or no effect on villus height in sham-irradiated mice.

**Figure 3:**
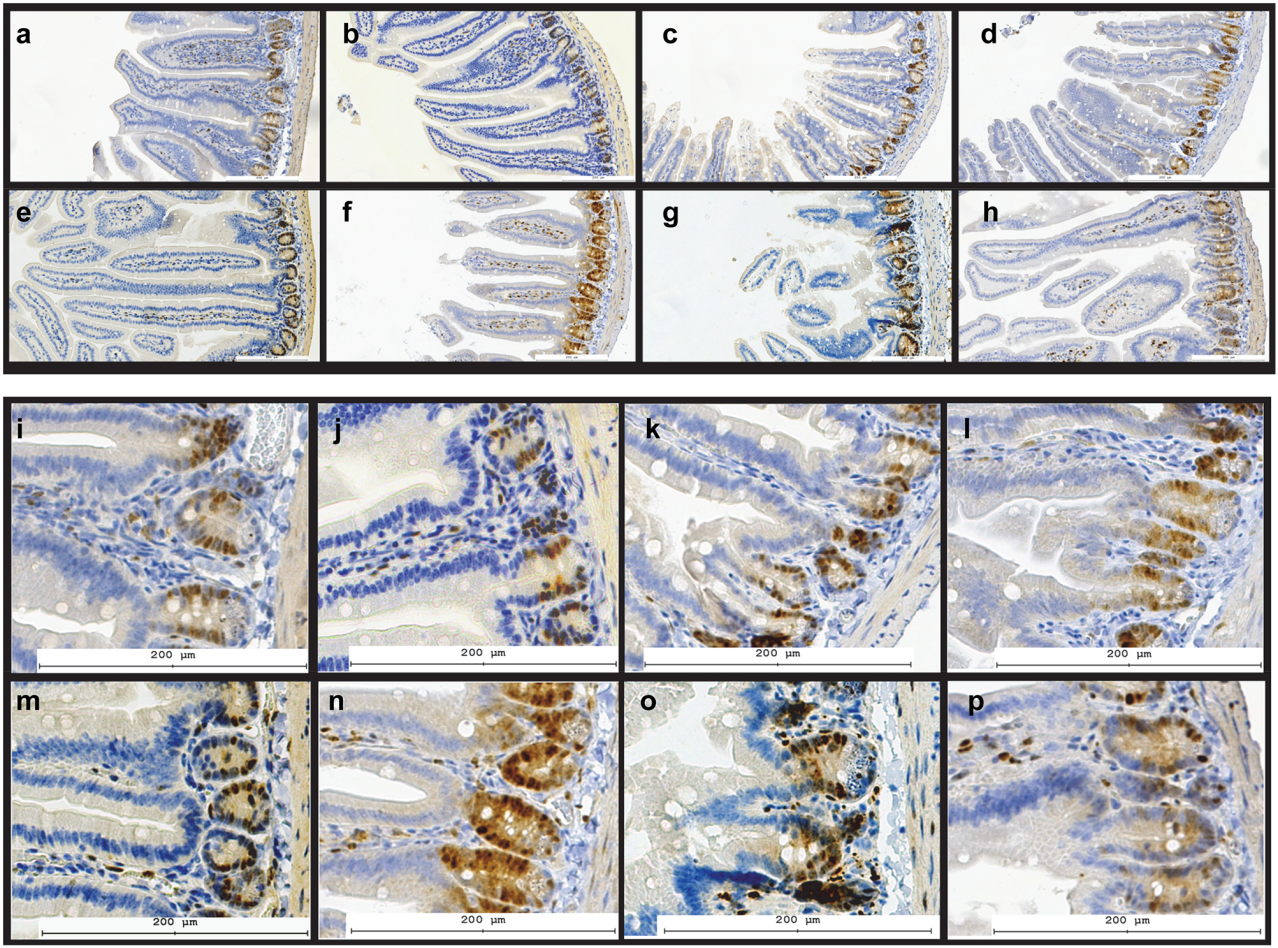
The effect of p*2TA* peptide on Cyclin D1 expression in mouse intestine. Expression of Cyclin D1 in mouse intestine: a–h) overview, i–p) details. a–d, i–l) A/J mice; e–h, m–p) BALB/c mice; a,e,i,m) sham irradiated controls; b,f,j,n) mice treated with p*2TA* peptide six days before tissue harvest; c,g,k,o) mice exposed to 8 Gy gamma rays total body irradiation seven days before the sacrifice; d,h,l,p) mice exposed to 8 Gy gamma rays that received 5 mg/kg p*2TA* peptide 24 hours after radiation exposure. In irradiated mice (c,g,k,o) villus integrity is disrupted, their crypts show disorganization, and very little Cyclin D1 staining can be observed outside the crypts. Intestines of irradiated mice that were treated with p*2TA* peptide 24 h later show a more normal morphology with respect to villus height and crypt appearance; they also show more numerous CD1 positive cells inside villi.

**Cyclin D1 expression** is a marker for cell proliferation in jejunal crypts [19], and is critical for the maintenance of tissue homeostasis. Figure 3 shows representative images of small intestine immunostained for Cyclin D1 protein. Quantification of Cyclin D1 staining showed that irradiated mice displayed significantly decreased Cyclin D1 staining in both BALB/c (31.7±10.6%, P<0.05) and A/J mice (59.7±9.2%, P<0.05), as compared to control animals. A decline in Cyclin D1 immunoreactivity was much less pronounced in irradiated BALB/c and A/J mice that received p*2TA* peptide, 79.3±16.8% (P<0.05; RP vs. R group) in BALB/c and 87.0±14.2% (P<0.05; RP vs. R group) in A/J mice. A single administration of p*2TA* peptide 24 h after whole-body irradiation exposure ameliorated radiation effect on Cyclin D1 expression in mouse intestine.

Radiation induced changes in villus morphology and organization of crypts are also noticeable in Figure 3; in irradiated and peptide treated mice Cyclin D1 stained GI sections resembled those in untreated animals.

**Figure 4:**
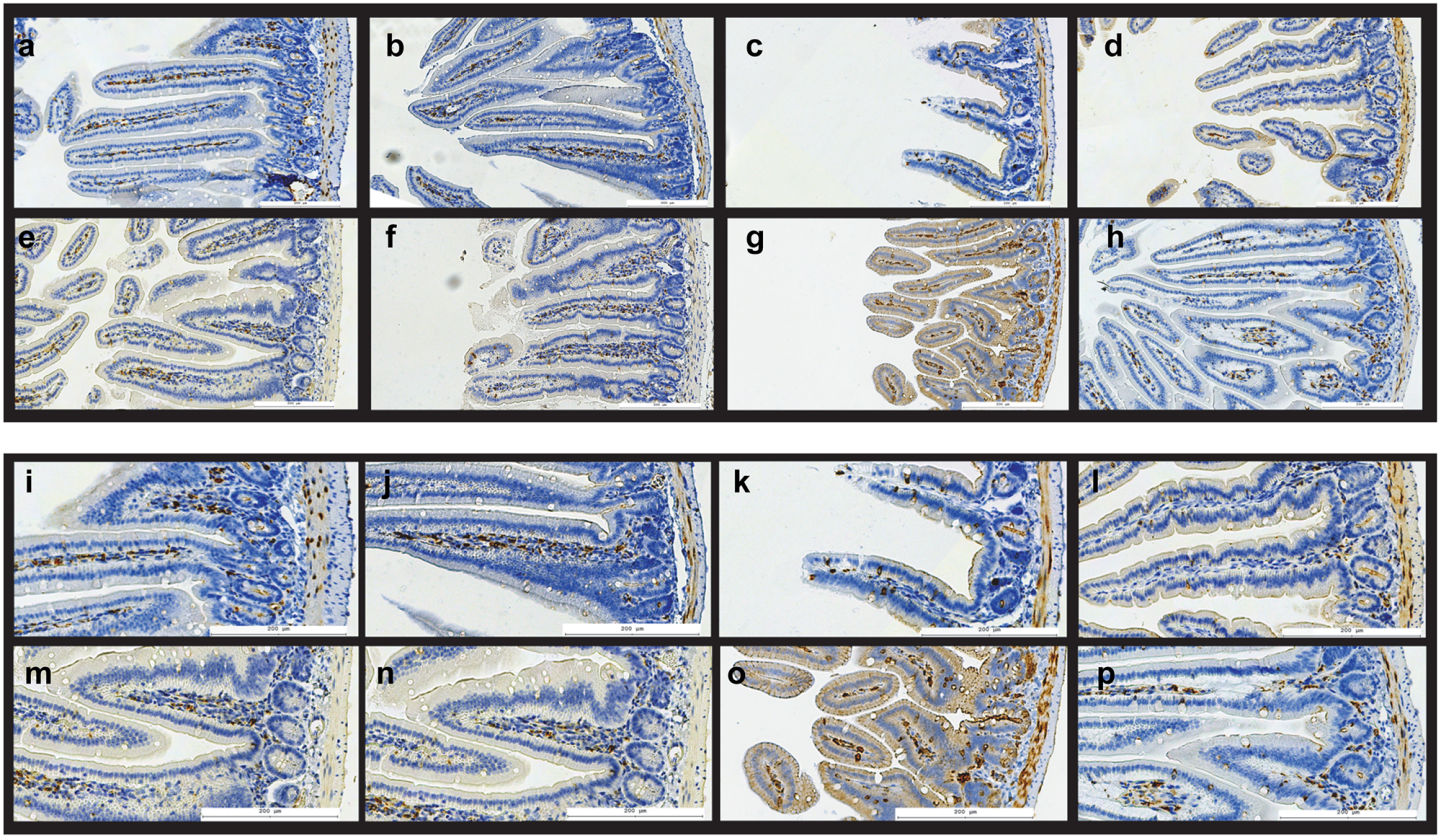
The effect of p*2TA* peptide on COX-2 expression in jejunal crypts. Expression of COX-2 in mouse intestine: a–h) overview, i–p) details. a–d, i–l) A/J mice; e–h, m–p) BALB/c mice; a,e,i,m) sham irradiated controls; b,f,j,n) mice treated with p*2TA* peptide six days before tissue harvest; c,g,k,o) mice exposed to 8 Gy gamma rays total body irradiation seven days before the sacrifice; d,h,l,p) mice exposed to 8 Gy gamma rays that received p*2TA* peptide 24 hours after radiation exposure. In irradiated mice (c,g,k,o) villus integrity is disrupted, their crypts show disorganization, and COX-2 staining is observable in cells in the surface layer of villi. Intestines of irradiated mice that were also treated with 5 mg/kg p*2TA* peptide 24 hours after irradiation show a more normal morphology with respect to villus height and crypt appearance, and they also show fewer COX-2 positive cells in villi surface cell layer, while COX-2 staining pattern inside villi replicates the pattern seen in sham irradiated mice.

**Cyclooxygenase-2** (COX-2) is a key inducible enzyme involved in prostaglandin production; it is generally undetectable in unperturbed epithelial tissues but can be strongly up-regulated by a number of inflammatory stimuli, including ionizing radiation [6], [20]. Figure 4 shows representative images of small intestine immunostained for COX-2 protein. Quantification of COX-2 positive staining in jejunal cross-sections has shown that irradiated group displayed significantly increased COX-2 immunoreactivity in both BALB/c (270.8±33.4%, P<0.05) and A/J mice (229.8±54.1%, P<0.05), as compared to control animals. Irradiated and peptide treated animals displayed much lower COX-2 immunoreactivity: 132.7±29.8% (P<0.05; RP vs. R group) in BALB/c and 142.7±28.5% (P<0.05; RP vs. R group) in A/J mice. Therefore, a single administration of p*2TA* peptide 24 h after radiation exposure ameliorated radiation effect on COX-2 expression in mouse intestine.

**Figure 5:**
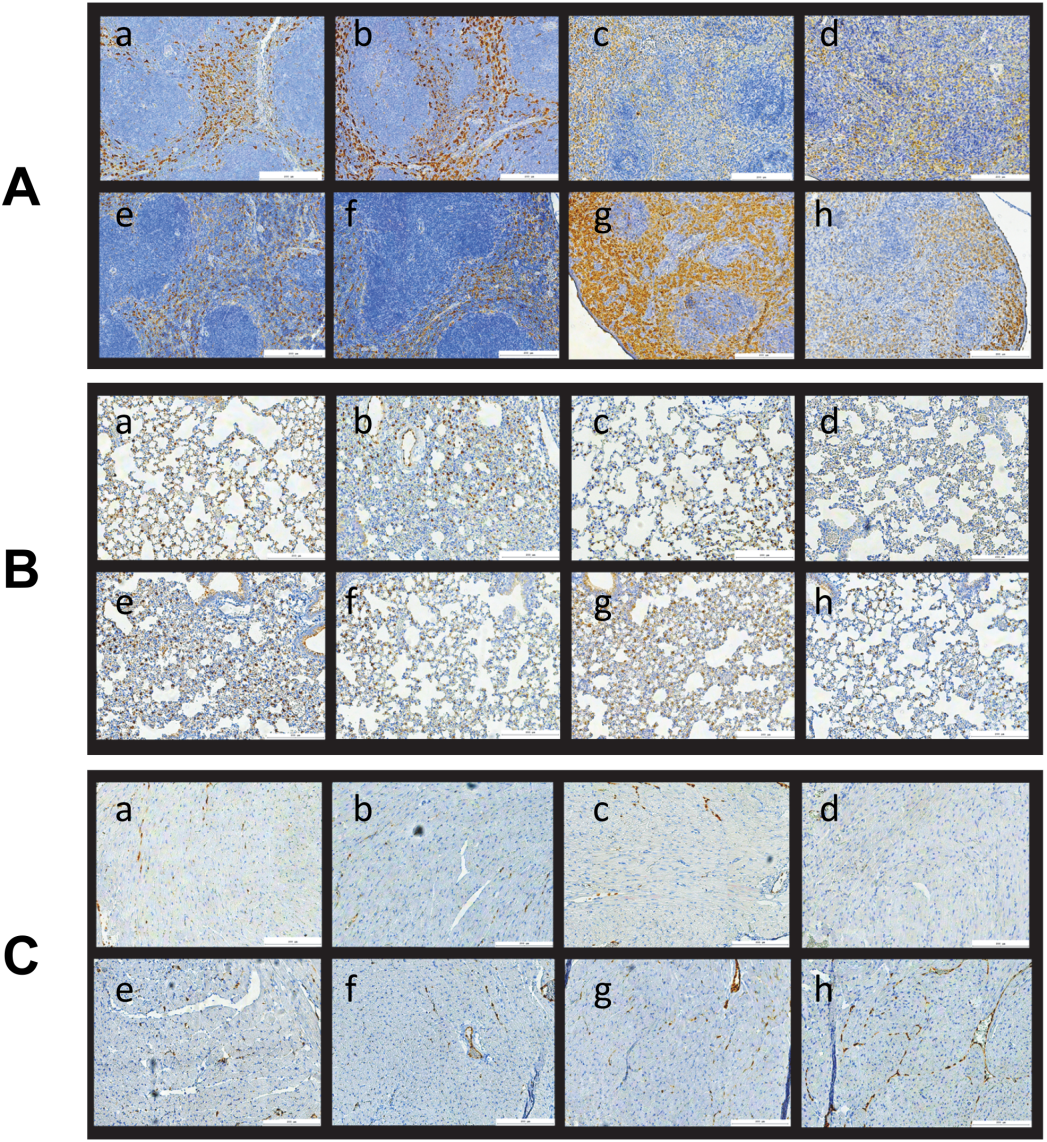
Immunohistochemistry of COX-2 in spleen, lung and heart. Spleen (**A**), lung (**B**) and heart (**C**) tissues were collected 7 days after total body irradiation and embedded in paraffin for immunohistochemistry staining. a–d) A/J mice; e–h) BALB/c mice; a,e) sham irradiated controls; b,f) mice treated with p*2TA* peptide 5 mg/kg six days before tissue harvest; c,g) mice exposed to 8 Gy gamma rays seven days before the sacrifice; d,h) mice exposed to 8 Gy gamma rays that received 5 mg/kg p*2TA* peptide 24 hours after radiation exposure. While irradiation increases COX-2 staining in spleen, its effect on lung and heart was not marked. Administration of p*2TA* peptide modulated the effect of irradiation on spleen samples.

Figure 5 shows lung, spleen and heart tissue samples immunostained for COX-2 protein; the effects of p2TA peptide on these tissues were found to be much less pronounced; on the other hand, apparent effects of irradiation on these tissues at the timepoint tested were not dramatic.

#### Macrophage recruitment to small intestine

**Figure 6:**
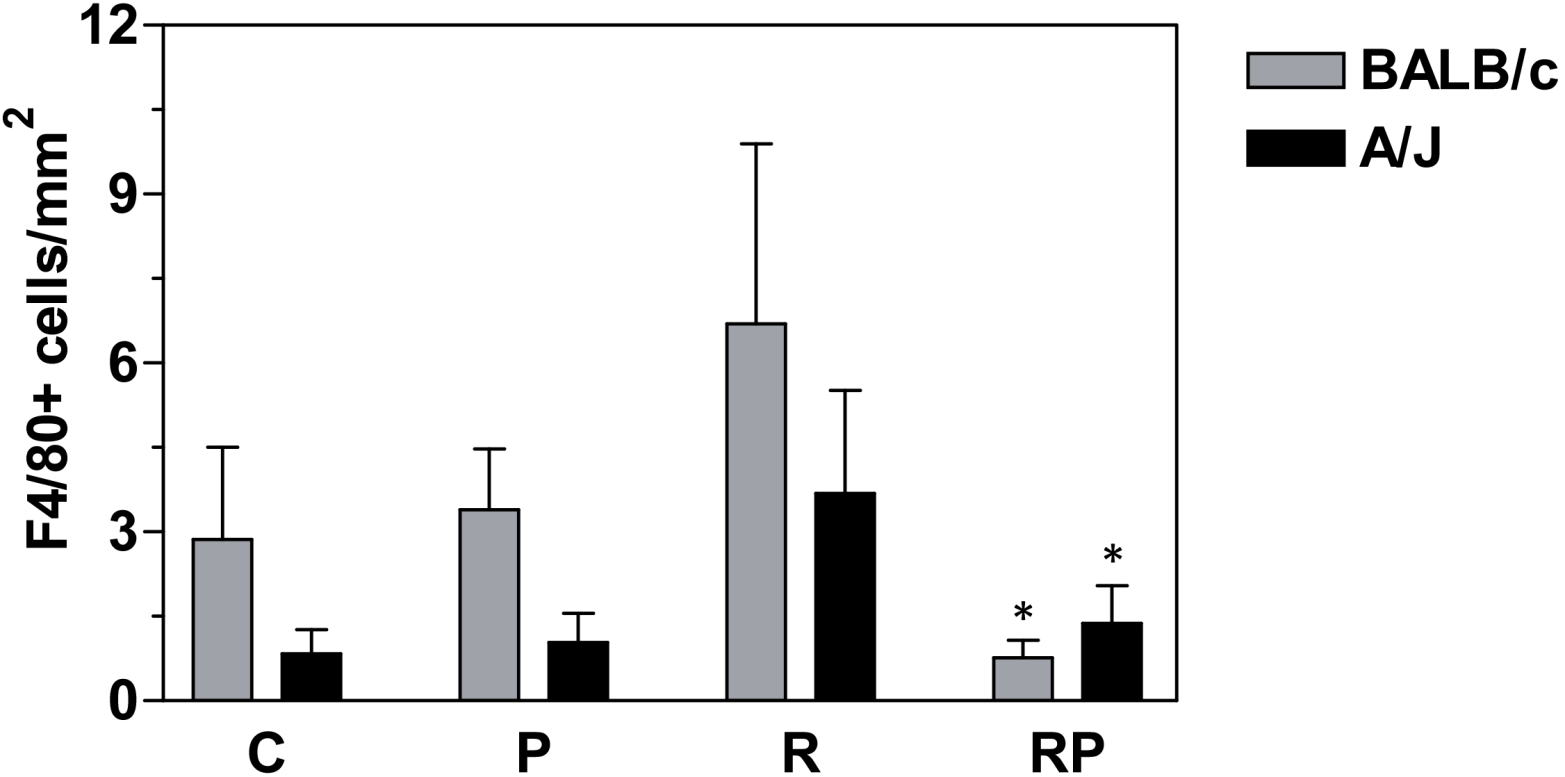
Immunohistochemistry of activated macrophage marker F4/80 in mouse jejunum. Jejunal tissues were collected 7 days after irradiation and embedded in paraffin. After fixation and processing, cross sections of jejunum were immunostained with F4/80 antibodies and number of F4/80 positive cells per mm^2^ area of tissue was counted using HistoQuest image analysis software. Bars represent the means ± SEMs for tissue sections of mice in each group. *Statistically significant differences between R and RP groups (P<0.05).

Increased presence of macrophages in irradiates tissues has been noted and associated with inflammatory response of tissues to ionizing radiation [6], [21], [22]. Activated macrophage marker F4/80 [23] was used for immunohistochemical staining of small intestine cross sections. As shown in Figure 6, samples from irradiated mice displayed increased presence of F4/80-positive cells, from 3.39±1.08 cells/mm^2^ in controls to 6.69±3.20 cells/mm^2^ in irradiated BALB/c mice (not a significant difference, C vs. R group) and from 1.04±0.52 cells/mm^2^ in controls to 3.68±1.84 cells/mm^2^ in irradiated A/J mice (P<0.05, C vs. R group). However, p*2TA* treated irradiated animals displayed decreased numbers of F4/80-positive cells in both strains of mice. Number of macrophages in RP group was 0.77±0.31 cells/mm^2^ (P<0.05, R vs. RP group) in BALB/c mice and 1.37±0.68 cells/mm^2^ (P<0.05, R vs. RP group) in A/J mice. Therefore, a single administration of p*2TA* peptide 24 h after radiation exposure compensated for radiation-induced increase in macrophage recruitment to mouse intestine.

## Discussion

This study is the first to demonstrate that the CD28-mimetic peptide p*2TA* mitigates many of the effects of ionizing radiation *in* *vivo*, both in the systemic circulation and in several tissues including the GI tract. Radiation-induced inflammation of the GI tract is considered to be one of the critical causes for systemic complications following radiation exposure, and may mediate some effects that lead to multiple organ failure [2], [3], [4]. Exposure to high doses of ionizing irradiation causes the loss of cells in jejunal crypts [13]. Apoptosis levels in crypt cells increase dramatically at 3 to 6 h after exposure to as little as 1 Gy of gamma-rays; on the other hand, an 8 Gy exposure leads to prolonged apoptosis observed as late as 60 h following exposure, although its peak occurs at earlier timepoints [24]. In addition to crypt stem cells, apoptosis of endothelial cells from the GI microvasculature is also considered critical for radiation caused GI toxicity [25].

Many different agents have been used to modulate *in* *vivo* effects of systemic or GI radiation injury, including a variety of cytokines and growth factors [1], [18], [25], some stimulating growth of intestinal stem cells, others endothelial cells of the microvasculature. Inhibition of COX2 was also suggested as a possible modulator of radiation responses [20]. More recent work has examined effects of genistein nanoparticles as radiation mitigators [26]. It is important to note, however, that in each case the proposed mitigating substance was administered either before or shortly after irradiation, or, alternatively, as a continuous, days long post-irradiation treatment. The work presented here is one of the very few where administration of the mitigator was done only once and as long as 24 h after exposure.

Short peptides including p*2TA* have been shown to attenuate the host’s inflammatory response by acting as modulators of CD28 signaling [7], [8], [9], [10]. This novel class of well-tolerated immunomodulators attenuates CD28 signaling in T cells but does not block it completely, while leaving the normal humoral immune response intact, and thus offers a unique approach for the treatment of infectious and inflammatory diseases. p*2TA* (AB103) was shown to be effective in the treatment of experimental toxic shock induced by superantigens [8], [9], in treatment of severe Gram-positive bacterial infection [9], and in a Phase 2a trial of patients with necrotizing soft tissue infections demonstrated substantial improvement across multiple clinical endpoints and was well tolerated [27]. Because CD28-oriented immunomodulators have been shown to be capable of regulating the host’s inflammatory responses, and because inflammation is one of the problems associated with radiation exposure, we hypothesized that these peptides may provide therapeutic benefit.

In this study, the octapeptide p*2TA* (AB103), a mimetic of the homodimer interface of CD28, was administered to two inbred strains of mice, BALB/c and A/J, 24 h after exposure to 8 Gy of IR. A series of systemic and tissue changes were anticipated as a result of IR. These included, at 7 days after irradiation, increased expression of IL-6, KC and fibrinogen in circulation [14], decreased viability of jejunal crypts, and increased inflammation associated with a new pattern of COX-2 expression in small intestine of IR mice [3], [11], [13], [24], [26]. In addition, increased presence of macrophages in irradiated mice was expected as well [5], [6]. All of these changes were observed in irradiated animals, and most of them were diminished in irradiated mice subsequently treated with anti CD28 peptide p*2TA*.

It should be noted that, while others found that the jejunal crypt cells of BALB/c mice were more radiosensitive than those of A/J animals [11], we found no big differences between the strains for the dose and timepoint used. Moreover, in this study plasma levels of IL-6 and KC cytokines were increased more in A/J than BALB/c mice (Figure 1), although this strain is believed to have a higher LD50/30 [12]. Cytokine production by immune system cells after exposure to ionizing radiation above LD50/30 can be expected to persist until the cells secreting them remain viable, therefore, it is possible that at 7 days after irradiation A/J mice may have more cytokine producing cells.

Peptide p*2TA* was given to irradiated mice 24 h after IR to investigate which of the features associated with IR exposure may be modulated because of the changes in immune system modulation. It is important to note that at that time point, for example, apoptosis of crypt stem cells has already reached its peak [24]. Statistically significant differences in the quantity of IL-6 and fibrinogen were noted, as well as a significant decrease in jejunal crypt toxicity induced by IR. In addition, p2TA mediated changes in numbers of F4/80 positive cells and expression patterns of COX-2 and Cyclin D1 in small intestine. It should be noted that Cyclin D1 expressing crypt cells produce anti-inflammatory glucocorticoids [19], which may lead to the development of a GI protective anti-inflammatory feedback loop. Nevertheless, probably the most prominent way in which *p2TA* exerts its activity is by downregulating CD28 signaling cascades of T cells. This surface antigen is critical for many of the pro-inflammatory lymphocyte activities, from interaction with CD68 on macrophages [23] to induction of suppression of excessive inflammatory response [8], [10].

These data suggest that modulation of the immune system responses following total body IR exposure acts as a potent mitigator of IR damage, matching in its potency many of the previously established treatments of IR injury. The beneficial effects of the CD28 mimetic p*2TA* (AB103) peptide in radiation-induced GI injury and systemic and tissue inflammation warrant further research in developing this and similar peptides as radiation mitigators following inadvertent exposures to total-body irradiation and as an aid in clinical trials in order to minimize radiation syndrome.
